# Identification of Factors Affecting Self-Efficacy in Women with Spontaneous Pregnancy Loss

**DOI:** 10.3390/healthcare11091217

**Published:** 2023-04-25

**Authors:** Mariola Mróz, Agnieszka Bień, Grażyna Iwanowicz-Palus, Justyna Krysa

**Affiliations:** Chair of Obstetrics Development, Faculty of Health Sciences, Medical University of Lublin, 4-6 Staszica St., 20-081 Lublin, Poland

**Keywords:** miscarriage, pregnancy loss, general self-efficacy, social support, quality of care

## Abstract

Pregnancy loss is a difficult situation that can affect a woman’s physical and psychological health, and thus requires appropriate management and support. An individual’s sense of self-efficacy is an important factor in the process of coping with a problem. Therefore, an analysis of self-efficacy in women after spontaneous pregnancy loss is warranted, so as to establish its association with social support, socio-demographic variables, quality of care, and specific behaviors of the medical staff. The cross-sectional study was performed in a group of 610 patients hospitalized due to spontaneous pregnancy loss in hospitals in Lublin (Poland). The study used a diagnostic survey with questionnaires: Generalized Self-Efficacy Scale (GSES), the Berlin Social Support Scales (BSSS), and a standardized interview questionnaire. Post-pregnancy loss patients rated partner support highest (M = 9.25), while the best-rated category of social support was perceived available instrumental support (M = 3.78). In relation to medical personnel, the quality of care provided by midwives was rated the highest (M = 4.57). The study demonstrated a statistically significant (*p* < 0.05) association between the selected socio-demographic factors and the specific types and sources of support on the one hand, and generalized self-efficacy on the other, in the patients after pregnancy loss who were studied. Socio-demographic factors that affected self-efficacy in the respondents included their relationship status and socio-economic standing. Self-efficacy is positively correlated with social support in women after pregnancy loss.

## 1. Introduction

The Royal College of Obstetricians and Gynecologists applies the term “miscarriage” to pregnancy loss which occurs until 24 weeks of gestation [1]. In Poland, a miscarriage is defined as the loss of pregnancy before the 22nd week of gestation or when the weight of the fetus does not exceed 500 g [1,2]. The loss of a loved one is among the most painful and most difficult life events. The experience of lost maternity, e.g., due to a miscarriage, may be equally intense. Pregnancy loss may change an individual’s priorities and opinions, and affects the whole family [3,4]. One factor that affects the process of coping with a difficult situation is the sense of self-efficacy, defined as an individual’s belief about their ability to achieve their objectives in a specific life situation [5,6,7]. Self-efficacy is also a behavioral self-regulation mechanism, which individualizes people in terms of emotional, motivational, and cognitive functioning [7,8].

A review of the scientific literature did not yield many reports from studies on the subject in the field of gynecology, though the topic itself is highly important, as self-efficacy has an impact on one’s health-related behaviors and on coping with stress and disease. Belief in one’s self-efficacy is positively correlated with maintaining a healthy distance, optimism, and satisfaction with life. Individuals with a strong sense of efficacy are more prepared to overcome difficulties. In turn, those with less self-efficacy do not believe in their own capabilities, tend to give up in the face of difficulty, and are unable to tackle challenges. A weak sense of self-efficacy also makes one more susceptible to stronger emotions, prone to focusing on one’s weaknesses, and likely to experience more anxiety [5,6,9,10,11]. Research in a variety of populations has demonstrated that self-efficacy has a positive impact, mediating between social support and individual behavior in coping with specific life situations; it also plays a role in preventing depression [12,13].

The concept of social support gained prominence in the 1970s, when individuals who had friends, surrounded themselves with family, and belonged to a variety of organizations were found to cope better with stress. Social support is defined as assistance given by an individual or provided to them in challenging situations. It supports one in achieving a specific objective or solving a problem [14]. Social support creates a relationship that provides an individual with the appropriate emotions or shows them the right courses of action. When an individual feels surrounded by people who can be relied on for support, this creates a sense of security and control in the face of crisis. Support is also a recognized factor that positively affects one’s well-being and their ability to cope with stress [15]. To be effective, the support process should be targeted, and the extent of assistance should match the recipient’s needs and expectations [14]. A lack of support or inadequate support may have negative psychological consequences for the woman [16,17,18], which is why the issue should be addressed in research and the appropriate guidelines for medical staff should be introduced. The available empirical reports on pregnancy loss mainly focus on clinical aspects, including the incidence of depression and anxiety, but we have found no studies detailing the specific support activities performed by healthcare professionals or the impact of social support on self-efficacy among patients hospitalized due to pregnancy loss.

The aim of research was to identification of factors affecting self-efficacy in women with spontaneous pregnancy loss.

The specific objectives of the study indicated the evaluation of whether there is a relationship with self-efficacy of such factors as social support, quality of care, and sociodemographic variables.

## 2. Materials and Methods

The study was performed between August 2016 and February 2019 among patients hospitalized due to spontaneous pregnancy loss (until 22 weeks of gestation) in selected hospitals in Lublin, Poland. The criterion for selecting a hospital was the third, the highest level of reference (ability to provide top-quality specialist care for mothers and newborns).

### 2.1. Study Design and Participants

Sampling was non-probabilistic. The study included all patients who had miscarried, were hospitalized during the analyzed time, and met the inclusion criteria. Inclusion criteria were as follows: age above 18 years, informed consent to participate, loss of a singleton pregnancy up to 22 weeks, no psychophysical disorders, and a normal clinical condition. Patients in a poor psychological condition, receiving psychotherapy or psychiatric treatment, were excluded from the study.

The self-efficacy was studied dependent variable. In turn, the independent variables included: age, education, residence, relationship status, professional activity, self-reported financial standing, gynecologist, midwife, psychologist, partner, family, friends, spiritual, patients at hospital after pregnancy loss, Berlin Social Support Scales.

#### 2.1.1. Data Collection

Respondents were informed of the study purpose and course, and notified that participation was voluntary and anonymous, and that results would be used exclusively for research purposes. Out of the 645 patients recruited for the study, 610 returned fully and correctly completed questionnaires, and were included in subsequent statistical analyses (5 women did not provide consent to participate, and 30 questionnaires were incomplete or incorrectly completed). The response rate was 94.57%. The survey questionnaire (Supplementary S1) was given to each patient on the last day of her hospitalization (between days 3 and 6), once medical personnel had confirmed that her treatment had been completed and her psycho-physical condition had stabilized.

The study was approved by the Bioethics Committee of Lublin Medical University (KE-0254/221/2016), as well as the managers and department heads in each hospital where the study was performed.

#### 2.1.2. Assessments

The study used a diagnostic survey with questionnaires. The following research instruments were used: the Generalized Self-Efficacy Scale (GSES), the Berlin Social Support Scales (BSSS), and a standardized interview questionnaire.

##### Instruments

- The Generalized Self-Efficacy Scale (GSES) (developed by R. Schwarzer and M. Jerusalem, adapted into Polish by Z. Juczyński) is an instrument measuring the strength of an individual’s belief in their capacity to overcome obstacles and difficulties. Respondents rate 10 statements on a scale of 1 to 4, where 1 stands for “disagree”, 2—“somewhat disagree”, 3—“somewhat agree”, and 4—“agree”. The total score reflecting a respondent’s generalized self-efficacy is converted into standardized sten units. Low self-efficacy is indicated by sten 1–4 (up to 24 points), moderate—by sten 5–6 (25–29 points), and high—by sten 7–10 (≥30 points). Cronbach’s α for the GSES scale is 0.85 [5,19].
- The Berlin Social Support Scales (BSSS) (by R. Schwarzer and U. Schulz, adapted into Polish by A. Łuszczyńska and M. Kowalska) are a set of independent instruments measuring the cognitive and behavioral dimensions of social support: perceived available emotional support, perceived available instrumental support, need for support, support seeking, actually received support, and protective buffering. The BSSS questionnaire contains 6 independent subscales that can be used together or separately. Respondents rate each item on a scale of 1 to 4, where 1 indicates complete disagreement, while 4 denotes complete agreement with the statement. Higher scores indicate more social support. In the full version of the questionnaire there is a scale that examines those who are close to the respondent. For the purposes of this study, only scales assessing the respondent’s social support were used (hence, the protective buffering subscale was not included in the study as it contains questions aimed at those providing support). In the present study, we used scales for perceived available instrumental and emotional support, actually received support, need for support, and support seeking. Results are reported as means for each scale. Cronbach’s α for the instrument is 0.80 [20].
- Authors’ own survey questionnaire, comprising items associated with the study subject and the studied women’s characteristics. The respondents rated the level of support from each source (obstetrician-gynecologist, midwife, psychologist, husband/partner, family members, friends, patients in a similar situation hospitalized at the same time) on a scale of 1 to 10, with 1 denoting completely inadequate support, and 10—entirely sufficient support. They also rated the quality of hospital care (provided by their physician, midwife, and psychologist) on a scale of 1 to 5, with 1 being the lowest score, and 5 being the perfect score. In addition, the respondents rated statements regarding tasks performed by medical staff (diagnostics and care) in the context of informational, emotional, and instrumental support on a 5-item Likert scale, with 1 meaning the task has definitely not been performed, and 5, that it definitely has been performed. Cronbach’s α for the instrument is 0.90.

### 2.2. Statistical Analysis

The collected study material was analyzed using the IBM SPSS Statistics package (version 21). Quantitative variables were described using mean (M), median (Me), standard deviation (SD), and minimum (Min) and maximum (Max) values. Qualitative variables were presented using numbers (*n*) and percentages (%) for each category. For comparisons between two independent groups, the Mann–Whitney U-test (Z) was used. For comparisons between more than two independent groups, the Kruskal–Wallis ANOVA on ranks (H) was used. For associations between quantitative variables, Pearson’s correlation coefficient (r) was used. The results were considered statistically significant at *p* < 0.05.

## 3. Result

Table 1 shows respondents’ characteristics. Most women were aged between 26 and 30 years (32.6%) had completed college/university education (61.1%), lived in province capitals (47.7%), were married (80.5%), performed white-collar work (48.7%), and reported a good socio-economic standing (60.8%). Furthermore, most of the women had planned their pregnancy (70.8%).

Mean generalized self-efficacy (GSES) score among the women after a miscarriage was 30.29 (SD = 4.01).

In terms of social support, the highest scores were noted for perceived available instrumental support (M = 3.78), and the lowest for support seeking (M = 3.09). Among the sources of support, the partner was rated highest (M = 9.25), while the psychologist was seen as providing insufficient support (M = 3.86).

For quality of care, the highest scores were attributed to the midwife (M = 4.57), and the lowest, again, to the psychologist (M = 2.33)—Table 2.

The study demonstrated a statistically significant association between the respondents’ marital status and socio-economic standing, on the one hand, and generalized self-efficacy, on the other. Women who were single (*p* = 0.002) and those with a very good socio-economic standing (*p* < 0.001) had higher GSES scores than those who were married or reported a poor socio-economic standing (Table 3).

There was a statistically significant (*p* < 0.05) positive correlation of perceived available instrumental (r = 0.147), emotional (r = 0.151), and currently received (r = 0.098) social support with women’s generalized sense of self-efficacy after miscarriage. In addition, a statistically significant (*p* < 0.05) negative correlation was found between support seeking (r = −0.086) and needing social support (r = −0.227) and sense of self-efficacy. There was also a statistically significant (*p* < 0.05) positive correlation between support from friends (r = 0.108) and other patients in a similar situation (r = 0.135) with the generalized sense of self-efficacy of the women studied.

An analysis of correlations between self-efficacy and the respondents’ views on the quality of care provided by medical personnel did not demonstrate statistically significant associations (*p* > 0.05)—Table 4.

Next, we verified whether selected activities performed by physicians and midwives have any effect on the sense of self-efficacy in women hospitalized after pregnancy loss. An analysis of our data demonstrated that the respondents’ sense of self-efficacy was significantly positively correlated with medical staff behaviors associated with informational support, such as: answering questions willingly (r = 0.105, *p* = 0.009), providing health-related information (r = 0.095, *p* = 0.019), preparing the patient for diagnostic and treatment procedures (r = 0.124, *p* = 0.002) and for post-discharge management (r = 0.120, *p* = 0.003), or notifying relatives about the patient’s needs and condition (r = 0.082, *p* = 0.042).

Our analysis also demonstrated an association between the medical staff’s performance of tasks related to emotional support and patients’ GSES scores. A statistically significant positive association (*p* < 0.05) was found for such behaviors as: expressing willingness to help (r = 0.131, *p* = 0.001), ensuring peace and quiet (r = 0.124, *p* = 0.002), considering the patient’s opinion (r = 0.111, *p* = 0.006), respecting her privacy (r = 0.113, *p* = 0.005), providing a sense of security (r = 0.093, *p* = 0.022), and showing interest in her well-being (r = 0.099, *p* = 0.014).

Likewise, the self-efficacy of women hospitalized after pregnancy loss was significantly positively correlated with selected diagnostic and care activities associated with instrumental support: providing rapid diagnostics so that the patient is not kept waiting (r = 0.112, *p* < 0.006), performing diagnostic and treatment procedures in a delicate manner (r = 0.141, *p* < 0.001), continuously observing the patient (r = 0.117, *p* = 0.004), adjusting the daily schedule to the patient’s needs if possible (r = 0.163, *p* < 0.001), responding to calls quickly (r = 0.125, *p* < 0.002), and allowing the patient’s family to assist in her care (r = 0.121, *p* = 0.003)—Table 5.

## 4. Discussion

In cases of miscarriage, the health problem is complicated by the added stress of pregnancy loss. The loss of a pregnancy is a highly stressful situation which may prompt a psychological crisis. It is associated with pain, hospitalization, social isolation, limitation in one’s social roles, and a reduced sense of security. Therefore, it is a time of increased need for social support [3,21,22,23,24,25]. A review of scientific literature did not yield many empirical reports from the field of gynecology concerning the subject matter addressed here, including self-efficacy, social support, and quality of care in women after pregnancy loss. Studies by specialists in other fields demonstrate that self-efficacy is among the factors that determine the process of coping with problems [5].

Self-efficacy is a significant factor in the development of positive psychological changes after a traumatic event (including coping with stress, reduction of depression symptoms, return to a state of psychological balance). The correlation may also take the opposite direction—trauma and PTSD could affect an individual’s self-efficacy (a tragic experience may strengthen one’s belief in their ability to survive and cope with difficult situations) [26].

Patients included in our study had a good level of generalized self-efficacy. Similar results were obtained in a group of post-mastectomy patients and in pregnant women with diabetes [5,27]. The situation was different in the patients with osteoporosis studied by Janiszewska et al. and the patients with physical disabilities studied by Byra, as both groups had a low level of this parameter [9,28].

An individual’s self-efficacy may be affected by individual personal characteristics, as well as socio-demographic factors. Information on the subject can be gleaned from a review of scientific literature, including findings from studies on a variety of patient groups by Janiszewska et al., Schwartz et al., Imes et al., and D’Souza et al. [9,24,25,27,28,29,30,31,32,33].

The generalized sense of self-efficacy of patients after pregnancy loss was influenced by marital status. Women who were unmarried or in an informal relationship and hospitalized with a diagnosis of pregnancy loss were more confident in their ability to cope with difficult and problematic situations. In a study by Rogala et al. involving respondents with reproductive organ cancer, patients living in a relationship had higher levels of self-efficacy than those living alone [30].

Perhaps this has to do with the age difference between the study groups and the additional social factor of today’s increasing trend toward non-marital relationships. It is also possible that this difference in the case of a specific group of patients after prenatal loss was influenced by an adaptation factor to the situation of becoming pregnant without being in a relationship—presumably, it could have been a mental preparation for single motherhood, before the miscarriage occurs.

Social support plays an important role in the progression through the subsequent stages of grieving, while inadequate support contributes to complicated grieving reactions [23]. A study conducted in a group of women after pregnancy loss also showed the impact of social support on their quality of life [2]. Respondents in our diagnostic survey reported the most support in terms of perceived available and actually received support, with the lowest scores for need for support and support seeking. Statistical analysis results are comparable to those reported by Iwanowicz-Palus et al. and Konczelska et al. [27,34].

For the purposes of our paper, we performed an analysis of correlations between specific sources of social support and the generalized sense of self-efficacy in women after pregnancy loss. Our findings indicate that higher levels of support from friends and other patients in a similar situation hospitalized at the same time are associated with higher levels of self-efficacy in the women studied. Though individuals close to the recipient of support may have an impact on the recipient’s self-efficacy [5], in our study, no association was found between the GSES score and the reported level of support from one’s partner, family members, or medical staff. Our findings thus diverged from those obtained in an analysis by Schwartz et al., who reported that multiparous women who rated their partners as “unsupportive” had significantly lower self-efficacy scores [29].

A special type of social interaction is the communication of medical personnel with patients after pregnancy loss. Patients rate the quality of care received during hospitalization in terms of accessibility, courtesy and communication, responsibility, and competence of the medical staff [35,36,37,38,39,40,41]. Our respondents hospitalized due to pregnancy loss reported very good quality of care received from the midwife and obstetrician-gynecologist. A large group of patients did not express an opinion on the quality of psychological care or rated it much lower than other members of the treatment team.

Psychological support is extremely important because of the emotional consequences of pregnancy loss. A psychologist can support women in building a sense of self-efficacy therefore ensuring contact with this professional is an important part of care in this patient group.

In our results obtained in a group of patients after pregnancy loss, there was no correlation between patients’ opinion regarding the quality of care received and self-efficacy. These findings diverged from those reported by Rogala et al., whose analysis demonstrated a high level of self-efficacy in patients of an obstetric ward and its association with their view of hospital care quality. In addition, the mothers of babies born in a better overall condition had a better view both of their self-efficacy and the quality of care received from medical staff [30]. In turn, the study by Schwartz et al. showed that in women with low levels of self-efficacy who received informational support, continuous care, and opportunities to discuss their beliefs and feelings, the sense of self-efficacy became stronger. When their stories were listened to, women were able to minimize the symptoms of trauma and to improve their attitude towards a subsequent labor [25]. Furthermore, those who received informational and emotional support during a subsequent pregnancy expressed satisfaction with this form of assistance [29,42,43,44,45,46]. In our study, in turn, specific behaviors of medical staff related to informational, emotional, and instrumental support were found to contribute to a stronger sense of self-efficacy in women after pregnancy loss.

When planning interventions in the field of care for women after pregnancy loss, as well as that health education and health promotion, it is important to include activities promoting patients’ self-efficacy and adaptation to their health situation, thus improving the quality of care and patients’ satisfaction with it. Care standards followed by hospital staff should not be based on their individual beliefs, but on the patient’s actual needs and expectations, as well as their personal capabilities of going through the adaptive process of coping with loss. The appropriate behaviors on the part of the treatment team may help optimize obstetric care and minimize any adverse outcomes in terms of the patient’s health.

### Strengths and Limitations of This Study

Studies on women after pregnancy loss are typically performed weeks or months after the event and are mainly clinical or psychological. Surveys for the present study were administered during hospitalization, as this is when most patients begin their coping process, which requires the medical staff to undertake the appropriate interventions from the outset. Our study used a standardized instrument, allowing other researchers in the area of pregnancy loss to compare results and monitor changes. The strengths of this study also include its large sample size and personal communication with each respondent.

Diagnostics and treatment procedures performed during hospitalization were a potential obstacle to the performance of the study, including the personal administration of the questionnaire, and therefore, to ensure the highest possible quality of the study, information on each patient’s clinical condition and treatment stage was obtained from medical personnel before contact with the patient. The study instrument was given to each patient on the last day of her hospitalization, having ascertained that her treatment had been completed and her psycho-physical condition had been stabilized. We are aware that a truly stable psychological condition can hardly be reached so soon after pregnancy loss, but each patient was contacted before the actual survey and provided with detailed explanations regarding the purpose and course of the study, including the voluntary nature of participation. The study is also limited by its cross-sectional nature, which does not allow for the establishment of cause-effect relationships between social support and self-efficacy, socio-demographic factors. The study is a continuation of the work in the field of pregnancy loss (from 2021).

## 5. Conclusions

Socio-demographic factors affecting the self-efficacy of women after spontaneous pregnancy loss include relationship status and socio-economic standing—single women and those reporting a very good socio-economic standing demonstrate higher levels of self-efficacy. In women having experienced pregnancy loss, self-efficacy is positively correlated with perceived available emotional and instrumental support, actually received social support, and sources of support including friends and other patients in a similar situation. Self-efficacy is negatively correlated with the need for support and support seeking. Specific behaviors of physicians and midwifes related to informational, emotional, and instrumental support are positively correlated with self-efficacy in women after pregnancy loss.

Our findings may be used to promote knowledge on appropriate ways to provide support, make care more professional, and develop training programs and standards for healthcare professionals in terms of providing care to patients in the difficult situation resulting from pregnancy loss.

## Figures and Tables

**Table 1:** Participants’ characteristics.

Participants’ Characteristics		*n*	%
Age	<25 y/o	77	12.6
	26–30 y/o	199	32.6
	31–35 y/o	195	32.0
	>35 y/o	139	22.8
Education	Primary education	39	6.4
	High school education	198	32.5
	College/university	373	61.1
Residence	Urban—province capital	291	47.7
	Urban—other	116	19.0
	Rural	203	33.3
Relationship status	Married	491	80.5
	Single/informal relationship	119	19.5
Professional activity	Professionally inactive	135	22.1
	White-collar work	297	48.7
	Blue-collar work	178	29.2
Self-reported financial standing	Very good	93	15.2
	Good	371	60.8
	Moderate	146	24.0
	Bad	6	1.0
Planned pregnancy	Yes	432	70.8
	No	178	29.2

**Table 2:** Ratings for social support dimensions and sources, hospital care quality and respondents’ self-efficacy.

	Score	M	SD	Me	Min	Max
	Generalized Self-Efficacy	30.29	4.01	30	17	40
Berlin Social Support Scales	Perceived available emotional support	3.68	0.44	3.75	1	4
	Perceived available instrumental support	3.78	0.43	4	1	4
	Need for support	3.16	0.57	3.25	1	4
	Support seeking	3.09	0.66	3.2	1	4
	Actually received support	3.6	0.4	3.8	1	4
Sources of support	Gynecologist	7.98	2.43	9	1	10
	Midwife	8.68	1.95	10	1	10
	Psychologist	3.86	3.69	1	1	10
	Partner	9.25	2	10	1	10
	Family	9.11	2	10	1	10
	Friends	8.51	2.6	10	1	10
	Spiritual	5.64	3.78	5	1	10
	Patients at hospital after pregnancy loss	7.79	2.99	9	1	10
Quality of care	Gynecologist	4.32	0.92	5	1	5
	Midwife	4.57	0.76	5	1	5
	Psychologist	2.33	1.6	1	1	5

(M)—mean, (Me)—median, (SD)—standard deviation, (Min)—minimum, (Max)—maximum.

**Table 3:** Analysis of associations between sociodemographic variables and self-efficacy in women after pregnancy loss.

Participants’ Characteristics		Generalized Self-Efficacy		Statistical Analysis
		M	SD	
Age	<20 y/o	30.32	4.13	H = 0.897 *p* = 0.639
	21–25 y/o	30.53	4.02	
	31–35 y/o	30.01	4.07	
	>35 y/o	30.31	3.88	
Education	Primary education	31.05	5.06	H = 1.586 *p* = 0.208
	High school education	30.12	3.92	
	College/university	30.29	3.94	
Residence	Urban—province capital	30.58	3.93	H = 1.321 *p* = 0.250
	Urban—other	30.17	4.11	
	Rural	29.93	4.06	
Relationship status	Married	30.01	3.79	Z = −3.174 *p* = 0.002
	Single	31.44	4.67	
Professional activity	Professionally inactive	29.95	4.04	H = 2.022 *p* = 0.155
	White-collar work	30.47	3.97	
	Blue-collar work	30.24	4.08	
Self-reported financial standing	Very good	31.95	3.93	H = 30.025 *p* < 0.001
	Good	30.37	4.02	
	Moderate	29.03	3.42	
	Bad	28.50	7.64	
Planned pregnancy	Yes	30.15	3.96	Z = −1.283 *p* = 0.199
	No	30.61	4.14	

(M)—mean, (Me)—median, (SD)—standard deviation

**Table 4:** Correlations between ratings for support dimensions and sources, quality of care, and respondents’ self-efficacy.

	Socio-Demographic Variables	Generalized Self-Efficacy	
		r	*p*
Berlin Social Support Scales	Perceived available emotional support	0.151	<0.001
	Perceived available instrumental support	0.147	<0.001
	Need for support	−0.227	<0.001
	Support seeking	−0.086	0.034
	Actually received support	0.098	0.015
Sources of support	Gynecologist	0.05	0.222
	Midwife	0.034	0.414
	Psychologist	0.084	0.158
	Partner	0.017	0.673
	Family	0.074	0.074
	Friends	0.108	0.011
	Patients at hospital after pregnancy loss	0.135	0.003
Quality of care	Gynecologist	0.048	0.242
	Midwife	0.07	0.086
	Psychologist	0.062	0.329

r—Pearson’s correlation coefficient.

**Table 5:** Correlations between ratings for tasks performed by the medical staff and respondents’ self-efficacy.

	Statements	Generalized Self-Efficacy	
		r	*p*
Informational support	They explained how I should prepare for diagnostic/treatment procedures	0.124	0.002
	They informed me about what to do after leaving the hospital	0.120	0.003
	They were willing to answer questions	0.105	0.009
	They provided complete information about my health	0.095	0.019
	They informed my relatives about my health and needs	0.082	0.042
	They provided information about diagnostic procedures	0.074	0.068
	They provided guidance and advice if necessary	0.070	0.082
	They provided information about support groups for pregnancy loss and other ways to get help	0.036	0.378
Emotional support	They expressed willingness to help	0.131	0.001
	They tried to ensure peace and quiet	0.124	0.002
	They respected my privacy	0.113	0.005
	They considered my opinion	0.111	0.006
	They were interested in what I was doing and how I was feeling	0.099	0.014
	The medical staff gave me a sense of security	0.093	0.022
	They were kind and respectful	0.062	0.123
	They allowed me to express my emotions	0.059	0.143
	They were delicate when giving me the news about the pregnancy loss	0.050	0.214
	They expressed sympathy	0.049	0.223
	They supported me with specific gestures	0.037	0.359
Instrumental support	They adjusted the daily schedule to my needs if possible	0.163	<0.001
	They performed all diagnostic/treatment procedures in a delicate manner	0.141	<0.001
	They responded to my calls quickly	0.125	0.002
	They allowed my family to assist in my care	0.121	0.003
	They continuously observed me and monitored my health	0.117	0.004
	They performed rapid diagnostics, so I was not kept waiting	0.112	0.006
	They provided painkillers if necessary	0.051	0.209

Instrumental support—diagnostics, care activities.

## Data Availability

Data are available upon reasonable request.

## Supplementary Materials

The following supporting information can be downloaded at: https://www.mdpi.com/article/10.3390/healthcare11091217/s1, Supplementary S1: SURVEY QUESTIONNAIRE.
