# Supplementary material for: Identification of Factors Affecting Self-Efficacy in Women with Spontaneous Pregnancy Loss
Source: Healthcare (Basel). 2023 Apr 25;11(9):1217. doi: 10.3390/healthcare11091217 (PMC10178273; doi:10.3390/healthcare11091217)
Supplement: Supplementary file 1 [file healthcare-11-01217-s001.zip › healthcare-2262584-supplementary.pdf]

SURVEY QUESTIONNAIRE

1. Age

a) < 25 y/o ☐
b) 26–30 y/o ☐
c) 31-35 y/o ☐
d) >35 y/o ☐

2. Education

a) Primary education ☐
b) Secondary education ☐
c) College/university ☐

3. Residence

a) Urban – province capital ☐
b) Urban – other ☐
c) Rural ☐

4. Relationship status

a) Married ☐
b) Single ☐

5. Professional activity

a) Professionally inactive ☐
b) White-collar work ☐
c) Blue-collar work ☐

6. Self-reported financial standing

a) Verygood ☐
b) Good ☐
c) Moderate ☐
d) Bad ☐

7. Planned pregnancy

a) No ☐
b) Yes ☐

8. Please rate the level of suport received from specific sources in the hospital by marking the iranswers on a 10-point scale. 1 means that the support was komplet ely insufficient and 10 comple tely sufficient.

**Sources of support**	**1**	**2**	**3**	**4**	**5**	**6**	**7**	**8**	**9**	**10**
a) Gynecologist	☐	☐	☐	☐	☐	☐	☐	☐	☐	☐
b) Midwife	☐	☐	☐	☐	☐	☐	☐	☐	☐	☐
c) Psychologist	☐	☐	☐	☐	☐	☐	☐	☐	☐	☐
d) Partner	☐	☐	☐	☐	☐	☐	☐	☐	☐	☐
e) Family	☐	☐	☐	☐	☐	☐	☐	☐	☐	☐
f) Friends	☐	☐	☐	☐	☐	☐	☐	☐	☐	☐
g) Patients in hospital after pregnancy loss	☐	☐	☐	☐	☐	☐	☐	☐	☐	☐

9. On a scale of 1 to 5, please rate the quality of care that you received during your hospitalization by marking your answer with an X.
> 1 denote spoor quality, 5 — perfect quality.

Quality of care	1	2	3	4	5
a) Gynecologist	☐	☐	☐	☐	☐
b) Midwife	☐	☐	☐	☐	☐
c) Psychologist	☐	☐	☐	☐	☐

10. Pleaseratethefollowingitemsregardingthecarethatyoureceivedduringyourhospitalization.

**Statements**	**1** strongly disagree	**2** disagree	**3** no opinion	**4** agree	5 strongly agree
They explained how I should prepare for diagnostic/treatment procedures	☐	☐	☐	☐	☐
They informed me about what to do after leaving the hospital	☐	☐	☐	☐	☐
They were willing to answer questions	☐	☐	☐	☐	☐
They provided complete information about my health	☐	☐	☐	☐	☐
They informed my relatives about my health and needs	☐	☐	☐	☐	☐
They provided information about diagnostic procedures	☐	☐	☐	☐	☐
They provided guidance and advice if necessary	☐	☐	☐	☐	☐
They provided information about support groups for pregnancy loss and other ways to get help	☐	☐	☐	☐	☐
They expressed willingness to help	☐	☐	☐	☐	☐
They tried to ensure peace and quiet	☐	☐	☐	☐	☐
They respected my privacy	☐	☐	☐	☐	☐
They considered my opinion	☐	☐	☐	☐	☐
They were interested in what I was doing and how I was feeling	☐	☐	☐	☐	☐
The medical staff gave me a sense of security	☐	☐	☐	☐	☐
They were kind and respectful	☐	☐	☐	☐	☐
They allowed me to express my emotions	☐	☐	☐	☐	☐
They were delicate when giving me the news about the pregnancy loss	☐	☐	☐	☐	☐
They expressed sympathy	☐	☐	☐	☐	☐
They supported me with specific gestures	☐	☐	☐	☐	☐
They adjusted the daily schedule to my needs if possible	☐	☐	☐	☐	☐
They performed all diagnostic/treatment procedures in a delicate manner	☐	☐	☐	☐	☐
They responded to my calls quickly	☐	☐	☐	☐	☐
They allowed my family to assist in my care	☐	☐	☐	☐	☐
They continuously observed me and monitored my health	☐	☐	☐	☐	☐
They performed rapid diagnostics, so I was not kept waiting	☐	☐	☐	☐	☐
They provided painkillers if necessary	☐	☐	☐	☐	☐
